# Report of 2 cases of progressive nodular histiocytosis

**DOI:** 10.1016/j.jdcr.2026.07.001

**Published:** 2026-07-07

**Authors:** Evelyn Itzamara Figueroa Saavedra, María Elisa Vega Memije, Miren Lorea Cárdenas Hernández, Sonia Toussaint Caire

**Affiliations:** a Division of Dermatology, “Dr. Manuel Gea González” General Hospital, México City, México; b Department of Dermatopathology, “Dr. Manuel Gea González” General Hospital, México City, México

**Keywords:** non-Langerhans cell histiocytosis, progressive nodular histiocytosis, storiform pattern

## Introduction

Non-Langerhans cell histiocytoses comprise a heterogeneous group of disorders characterized by dermal accumulations of cells derived from macrophages, as well as cells with dendritic differentiation. Most of these conditions are rare.^1^, ^2^, ^3^

Progressive nodular histiocytosis is a subtype of xanthogranuloma and belongs to the group of non-Langerhans cell histiocytoses. It was first described in 1978 by Taunton *et al* in a 9-year-old girl.^4^^,^^5^

Although the disease can occur at any age, its incidence is higher in young adults, particularly during the third and fourth decades of life, with no predilection for either sex.^3^^,^^4^

Clinically, it is characterized by multiple yellow-brown papules and nodules of variable size, predominantly involving the head, neck, trunk, and extremities; mucosal involvement may also occur. The lesions typically follow a progressive course without spontaneous resolution.^4^^,^^6^

## Case 1

A 60-year-old man residing in Mexico City, who worked as a car attendant and had no relevant past medical history, presented with an 8-year history of a periocular lesion at the left lateral canthus. Over time, the lesion progressively increased in size and disseminated to the chin, neck, axilla, and right elbow. The patient sought medical attention because of mechanical visual obstruction caused by the periocular lesion.

Physical examination revealed a disseminated polymorphic dermatosis involving the head, trunk, and upper extremities. Lesions were located on the left lateral canthus, chin, axilla, and right elbow, and consisted of multiple papules and nodules of variable size, the largest measuring 3 × 2 cm. The largest lesion was dark brown, while the remaining lesions were yellow with well-defined borders and firm consistency. Dermoscopic examination demonstrated areas with a homogeneous yellow background and a brown-gray pseudonetwork (Fig 1).

**Fig 1:**
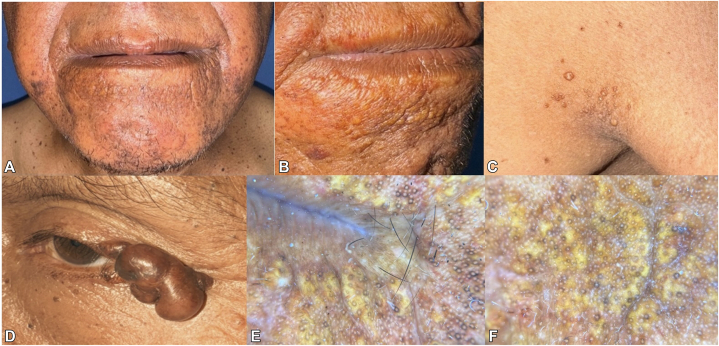
Multiple papules and nodules of variable size are observed **(A-C)**, with the largest lesion located in the left periocular region measuring 3 × 2 cm and displaying yellow and dark brown coloration **(D)**. Dermoscopic examination reveals areas with a homogeneous yellow background and a brown-gray pseudonetwork **(E** and **F)**.

Laboratory studies were obtained and were within normal limits, including triglyceride and cholesterol levels.

Three excisional biopsies were performed from the left lateral canthus, chin, and axilla. Histopathologic examination revealed, from the papillary dermis to the deep reticular dermis, a proliferation of spindle-shaped histiocytic cells arranged in a storiform pattern. The cells exhibited pale cytoplasm, with some containing multiple intracytoplasmic vacuoles of xanthomatous appearance, along with the presence of Touton giant cells, interspersed with thickened collagen.

Immunohistochemical analysis demonstrated positivity for CD68 and negativity for CD1a and Langerin (Fig 2).

**Fig 2:**
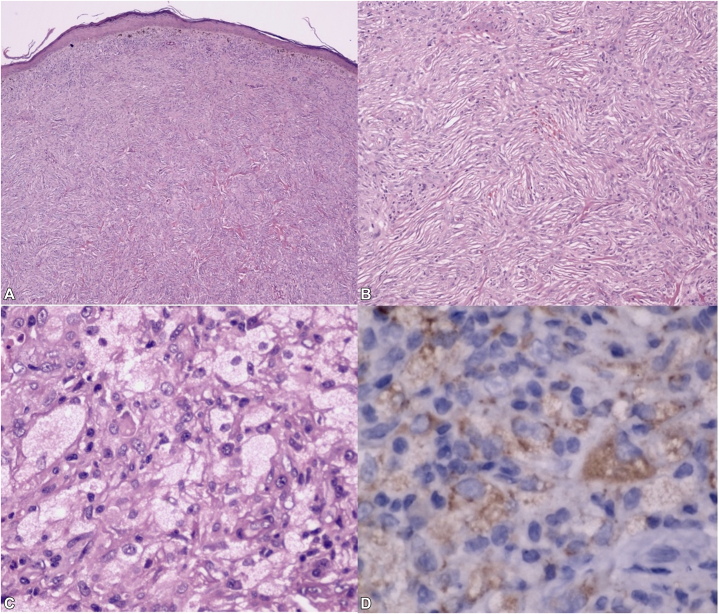
Histologic section stained with hematoxylin and eosin. From the papillary dermis to the deep reticular dermis, there is a proliferation of spindle-shaped histiocytic cells arranged in a storiform pattern **(A** and **B)**. The cells exhibit pale cytoplasm, with some containing multiple intracytoplasmic vacuoles of xanthomatous appearance, along with the presence of Touton giant cells, interspersed with thickened collagen **(C)**. Immunohistochemistry demonstrates CD68 positivity **(D)**.

A diagnosis of progressive nodular histiocytosis was established. To exclude mucosal involvement, upper endoscopy, colonoscopy, and laryngoscopy were performed, all of which showed no evidence of lesions. Treatment was initiated with methotrexate 15 mg weekly along with surgical excision of the left lateral canthus lesion.

## Case 2

A 52-year-old woman, originally from and residing in Veracruz, who worked as a high school teacher, with a medical history of type 2 diabetes mellitus and systemic arterial hypertension, presented with a 5-month history of cutaneous lesions on the scalp, with subsequent dissemination to the retroauricular region, tongue, and chest, accompanied by pain.

Physical examination revealed a disseminated dermatosis involving the head and trunk, affecting the scalp, retroauricular region, tongue, chest, and axillae. The lesions consisted of multiple papules and nodules of variable size, measuring approximately 3-4 mm, light in color, with some exhibiting a translucent, well-demarcated yellow appearance (Fig 3).

**Fig 3:**
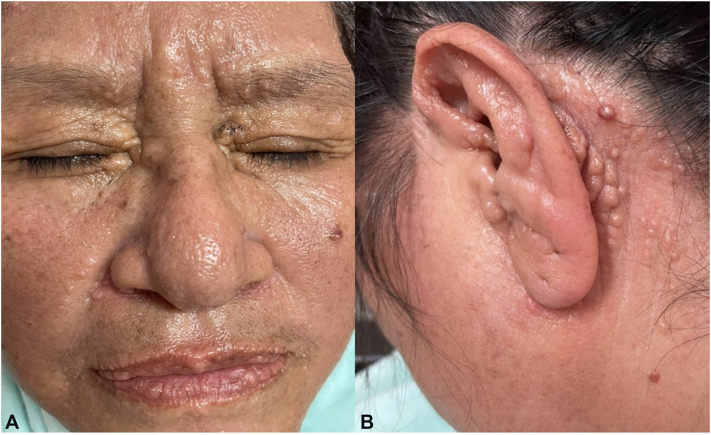
Disseminated dermatosis involving the head, affecting the face **(A)** and bilateral retroauricular regions **(B)**, characterized by well-demarcated yellow papular and nodular lesions measuring approximately 3-5 mm.

Laboratory studies were obtained and were within normal limits. An excisional biopsy of a scalp lesion was performed. Histopathologic examination revealed, from the papillary dermis to the deep reticular dermis, a proliferation of spindle-shaped histiocytic cells with clear, xanthomatous cytoplasm arranged in sheets (Fig 4). Immunohistochemistry demonstrated positivity for CD68. Upper endoscopy revealed multiple nodular lesions involving the esophageal mucosa. PET-CT imaging was not performed, and molecular studies, including BRAF mutation analysis and targeted next-generation sequencing, were not available, representing a limitation of this report.

**Fig 4:**
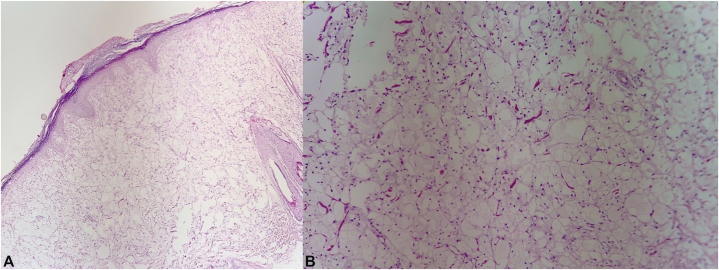
The entire dermis is occupied by spindle-shaped cells with clear, xanthomatous cytoplasm. Hematoxylin and eosin stain, original magnification ×10 **(A)** and higher magnification ×20 **(B)**.

## Discussion

Progressive nodular histiocytosis belongs to a subgroup of NLCH, within the juvenile xanthogranuloma family, which also includes xanthoma disseminatum, benign cephalic histiocytosis, adult xanthogranuloma, and generalized eruptive histiocytosis.^6^ Only a limited number of cases have been reported in the literature.

Clinically, progressive nodular histiocytosis is characterized by the progressive development of superficial yellow-brown papules and deeper reddish-brown nodules predominantly involving the head, trunk, and extremities. Mucosal involvement has been described in the oral, laryngeal, pharyngeal, and conjunctival mucosa.^1^ In our patients, Case 1 showed no mucosal involvement, whereas Case 2 demonstrated esophageal mucosal lesions.

Lesions typically do not regress spontaneously and progressively increase in number and size over time, potentially leading to marked cosmetic deformity. Facial involvement may produce a leonine appearance, and periocular lesions may result in ectropion or visual impairment.^6^^,^^7^ In Case 1, the patient presented with a periocular lesion causing mechanical visual obstruction without ectropion.

Laboratory studies typically reveal normal serum lipid levels.

Histopathologically, papular lesions usually demonstrate dermal infiltrates composed of histiocytes with abundant vacuolated cytoplasm and Touton giant cells, whereas nodular lesions are characterized by spindle-shaped histiocytes arranged in a storiform pattern.^4^^,^^6^ Immunohistochemistry typically demonstrates positivity for CD68 and factor XIIIa and negativity for CD1a, langerin, and S-100 protein.^3^

In 2011, Nofal et al proposed diagnostic criteria for progressive nodular histiocytosis, which include: the presence of 2 distinct types of generalized cutaneous lesions with a progressive clinical course, histopathologic findings compatible with xanthogranuloma, and an immunohistochemical profile consistent with non-Langerhans cell histiocytosis, characterized by CD68 positivity and negativity for CD1a and S100 protein. The absence of Birbeck granules on electron microscopy was also included among the criteria. Notably, the defining hallmark of progressive nodular histiocytosis is the coexistence of 2 clinically and histologically distinct lesion types.^6^

The main differential diagnosis of progressive nodular histiocytosis includes other forms of non-Langerhans cell histiocytosis within the xanthogranuloma group, which often share similar histologic and immunohistochemical features.^8^ (Table I)

**Table I:** Differential diagnoses

Disease	Clinical features
Generalized eruptive histiocytosis	Onset in adulthood; lesions appear on the trunk and proximal extremities. Characterized by red-bluish papules. New lesions occur in crops while older lesions resolve spontaneously.
Juvenile xanthogranuloma	Lesions usually appear before 6 y of age and resolve spontaneously within 2-3 y.
Xanthoma disseminatum	Onset between 5-25 y of age, with multiple brown-yellow nodules on the skin and mucous membranes, predominance in flexural areas, tendency toward spontaneous involution, and possible visceral involvement with lipid abnormalities.
Multicentric reticulohistiocytoma	Affects the head, neck, and trunk; presents as large, deep nodules over joints. Lesions may involute spontaneously. Articular involvement is characterized by progressive, mutilating destruction.
Benign cephalic histiocytosis	Multiple cutaneous lesions in the head and neck region in young children.

Recent evidence supports that progressive nodular histiocytosis should be considered a clonal disorder. Recurrent somatic mutations affecting the MAPK pathway, including alterations in KRAS and BRAF, have been identified in histiocytic disorders. This paradigm shift has important implications for diagnosis, classification, and treatment.

Histiocytoses are neoplasms primarily driven by activation of the MAPK pathway, secondary to mutations in genes such as MAP2K1, MAPK1, and BRAF. This molecular alteration has enabled the development of targeted therapies with favorable outcomes.^7^

MEK inhibitors, such as cobimetinib, have demonstrated high response rates even in cases without BRAF mutations, while in patients with BRAF V600E, agents such as vemurafenib have shown significant efficacy. Additionally, multikinase inhibitors such as regorafenib represent a therapeutic alternative by targeting multiple components of the MAPK pathway.^9^

There is currently no effective curative treatment for progressive nodular histiocytosis. Large and disfiguring lesions are often surgically excised; however, they frequently recur. Cryotherapy, carbon dioxide laser, and excimer laser have been used for the ablation of cutaneous nodules, although recurrence within surgical scars has been reported. Systemic therapies such as cyclophosphamide, vincristine, methotrexate, prednisolone, and baricitinib have also been described.^10^

Corticosteroid therapy has been reported in the form of intralesional injections, pulse therapy, and continuous daily treatment.^8^

Oral methotrexate 15 mg once weekly has been reported as successful in one case, with complete resolution of lesions and symptoms after 6 months. The patient remained in remission for 18 months while on therapy; however, recurrence was observed after discontinuation. Treatment was reintroduced, achieving remission again within 3 months.^11^

In a patient with laryngeal involvement, nodules were successfully removed using laser therapy.^12^

Both of our patients are currently under clinical surveillance, and Case 1 is being treated with methotrexate.

## Conflicts of interest

None disclosed.
